# A Linkage Map and QTL Analysis for Pyrethroid Resistance in the Bed Bug *Cimex lectularius*

**DOI:** 10.1534/g3.116.033092

**Published:** 2016-10-12

**Authors:** Toby Fountain, Mark Ravinet, Richard Naylor, Klaus Reinhardt, Roger K. Butlin

**Affiliations:** * Department of Biosciences, University of Helsinki, Finland; † Department of Animal and Plant Sciences, University of Sheffield, UK; ‡ Ecological Genetics Division, National Institute of Genetics, Mishima, Japan; § Department of Marine Sciences, University of Gothenburg, Sweden

**Keywords:** ectoparasite, household pest, insecticide, RAD-seq

## Abstract

The rapid evolution of insecticide resistance remains one of the biggest challenges in the control of medically and economically important pests. Insects have evolved a diverse range of mechanisms to reduce the efficacy of the commonly used classes of insecticides, and finding the genetic basis of resistance is a major aid to management. In a previously unstudied population, we performed an *F*_2_ resistance mapping cross for the common bed bug, *Cimex lectularius*, for which insecticide resistance is increasingly widespread. Using 334 SNP markers obtained through RAD-sequencing, we constructed the first linkage map for the species, consisting of 14 putative linkage groups (LG), with a length of 407 cM and an average marker spacing of 1.3 cM. The linkage map was used to reassemble the recently published reference genome, facilitating refinement and validation of the current genome assembly. We detected a major QTL on LG12 associated with insecticide resistance, occurring in close proximity (1.2 Mb) to a carboxylesterase encoding candidate gene for pyrethroid resistance. This provides another example of this candidate gene playing a major role in determining survival in a bed bug population following pesticide resistance evolution. The recent availability of the bed bug genome, complete with a full list of potential candidate genes related to insecticide resistance, in addition to the linkage map generated here, provides an excellent resource for future research on the development and spread of insecticide resistance in this resurging pest species.

The common bed bug, *Cimex lectularius* L. (Heteroptera, Cimicidae), is re-emerging as a significant economic and public health pest, precipitated by a recent global resurgence in populations (Boase 2001; Doggett and Russell 2008; Potter *et al.* 2008; Richards *et al.* 2009). Much of its recent success has been attributed to widespread resistance to insecticides (Romero *et al.* 2007; Romero and Anderson 2016), making pest control increasingly challenging and costly (Koganemaru and Miller 2013). Developing a more detailed understanding of the genetic and molecular basis of insecticide resistance is therefore of clear importance.

Previously, two point mutations, V419L and L925I, have been identified in the α-subunit gene of the voltage sensitive sodium channel (VSSC) that are functionally associated with resistance to the pyrethroid deltamethrin (Yoon *et al.* 2008). Pyrethroids are one of the most widely used insecticides, but, as over 80% of sampled populations in the United States (Zhu *et al.* 2010), and >95% of sampled populations in Europe (Booth *et al.* 2015), contained the V419L and/or L925I mutation(s), it is likely that target-site-based pyrethroid resistance has become widespread. In addition, several candidate loci associated with metabolic and penetrative resistance have been identified in studies comparing resistant and nonresistant populations, with increased expression of genes coding for detoxifying metabolic enzymes (including P450s, glutathione-S-transferases, and carboxylesterases), ATP-binding cassette (ABC) transporters and cuticular protein genes associated with pyrethroid resistance (Adelman *et al.* 2011; Mamidala *et al.* 2011, 2012; Zhu *et al.* 2012; Koganemaru *et al.* 2013).

The recent availability of the bed bug genome (Benoit *et al.* 2016; Rosenfeld *et al.* 2016) gives an ideal opportunity to further investigate the genetic basis of resistance. For example, 58 genes and one pseudogene coding for P450 enzymes have been identified in the *C. lectularius* genome (Benoit *et al.* 2016), with four of these genes previously implicated in pyrethroid resistance (Zhu *et al.* 2012). The further identification of genes coding for other metabolic enzymes, cuticular protein genes, and ABC transporters, allows the assessment of their contribution to resistance.

Although these genetic association and genome annotation studies have pointed to a promising group of candidate genes for pyrethroid resistance, their correlative top-down approach lacks the ability to demonstrate a direct association between any of these genes and the resistance trait. In addition, most of these studies used only one susceptible strain. Here, we perform an *F*_2_ mapping cross between a pyrethroid resistant and a susceptible bed bug population using RAD-sequencing. Our reduced-representation sequencing approach offers two advantages. First, we are able to reassemble >65% of the bed bug reference genome into 14 Linkage Group (LG)—a valuable resource for the community in future genome-based applications. Second, we are able to identify a new QTL associated with pyrethroid resistance that strongly implicates a functional role for a carboxylesterase encoding gene in this resistance trait.

## Materials and Methods

### Experimental cross design and phenotyping

An *F*_2_ mapping cross was established through mating a pyrethroid resistant female from a field population, originating from London (UK), with a pyrethroid susceptible male from a laboratory stock population, originating from a culture from the London School of Hygiene and Tropical Medicine [more information on these populations, called Field UK and Lab Stock, is available in Fountain *et al.* (2015)]. The field population was checked for the resistance phenotype prior to crossing to ensure resistance had not been lost. Our experimental design for QTL analysis with a single family assumes that the grandparents used to initiate the cross were homozygous for QTL involved in insecticide resistance, and for loci linked to these genomic regions. Because the lines were not highly inbred, this may not have been the case for all loci. However, since natural populations tend to have low heterozygosity (Fountain *et al.* 2014) and the lines had been maintained in the laboratory for multiple generations, it is likely that they were homozygous at resistance loci, and for the great majority of markers. One male and one female from the *F*_1_ offspring were selected at random and mated; 90 *F*_2_ offspring, along with the *F*_1_ parents, were subsequently phenotyped for pyrethroid resistance.

Pyrethroid resistance was tested using 40 mg/m^2^ of alpha-cypermethrin (Sigma number: 45806-100MG). The insecticide was dissolved in acetone and pipetted onto Whatman 90 mm Grade 1 cellulose filter paper (Sigma number: Z240079). Once the filter paper was dry, it was placed in a 90 mm diameter sterile polystyrene Petri dish. Individuals were added in groups (≤ 10 individuals per trial) and knock-down/mortality was scored at 24 and 48 hr. Phenotyping was performed at 26 ± 1° and 70 ± 5% relative humidity, with the knockdown/mortality score at 48 hr after exposure used as the resistance phenotype. Individuals were scored as susceptible (knocked down, unable to right themselves if turned over), partially resistant (able to right themselves, but walk with some difficulty), or resistant (walk normally, motor control apparently unaffected).

### DNA isolation and sequencing

Full body extractions (minus the head) were performed using DNAeasy Blood & Tissue Kit (Qiagen). RAD library preparation was performed as in Baird *et al.* (2008), using *Sbf*1. Following library preparation, sequencing was performed on a single Illumina HiSeq lane (100 bp PE) at the Natural Environment Research Council Biomolecular Analysis Facility at the University of Edinburgh, UK.

### Quality filtering and reference mapping

Following sequencing, library quality was checked using FASTQC (Babraham Informatics; http://www.bioinformatics.babraham.ac.uk/projects/fastqc). All downstream handling of sequencing data, with the exception of mapping to the reference genome, was conducted using the Stacks (v 1.35) pipeline (Catchen *et al.* 2011, 2013). Based on the average quality scores per read generated by FASTQC, the Stacks *process_radtags* module was used to remove any read where Phred quality scores fell to < 5 (*i.e.*, 3% error rate) in a 5 bp window. The module was additionally used to remove reads with traces of adapter sequence, remove any reads with an uncalled base, and demultiplex the pooled libraries. Following this initial processing, the *clone_filter* module was used on the paired-end sequence data for each individual in order to remove PCR duplicates—a major source of potential bias for RAD-sequencing approaches (Andrews *et al.* 2014).

Paired-end sequence reads filtered for duplicates were then aligned to one of the recently published *C. lectularius* genomes (Clec_1.0; NCBI accession number: PRJNA167477; Benoit *et al.* 2016) using GSNAP 2014-12-29 (Wu and Nacu 2010). We allowed a maximum of 10 alignments per read, no terminal alignments, and only the optimal hit to be reported. A maximum of four single nucleotide mismatches was allowed for each alignment.

### Stacks catalog construction and SNP calling

Aligned read data were processed using the reference-mapping branch of the Stacks pipeline (*ref_map.pl*), specifying an *F*_2_ cross, and identifying both parents and offspring using the –p and –s flags, respectively. We allowed a minimum of three reads to form a stack in the *pstacks* module (*i.e.*, minimum read depth for an allele, not a locus), and a single mismatch among loci during catalog construction. Stacks construction was conducted with these values following sensitivity testing with both *de novo* and reference mapping pipelines, revealing them to be the optimal parameters. SNPs were called using the Stacks default SNP calling method, *i.e.*, maximum likelihood estimation based on a multinomial probability distribution derived from the nucleotide frequency at each read position (Catchen *et al.* 2013). Following initial catalog construction, we used the *rxstacks* module to reanalyze and correct the *de novo* assembly. We filtered confounded loci (*i.e.*, loci within individuals matching multiple catalog loci, indicative of repetitive regions), pruned excess haplotypes (*i.e.*, removed potential erroneous haplotypes based on frequency), and recalled SNPs using Stacks’ bounded error model with ε = 0.1. Following catalog correction, the *genotypes* module was used to export SNP data from the main catalog. We used the module to perform automatic corrections to the data, and we only exported markers where at least eight *F*_2_ progeny had genotype calls. To clarify, this cutoff did not represent our final threshold for missing data, but was chosen to maximize the output loci for downstream filtering. Output genotype calls were filtered to include only loci that were heterozygous in the *F*_1_ parents (*i.e.*, AB/AB, where A and B are alleles from the female and male grandparents, respectively), then converted to R/qtl format using a custom R script (R Development Core Team 2015).

### Genetic map construction and QTL mapping

To perform QTL analysis, we used the R/qtl package (Broman *et al.* 2003). Our first step was to perform additional data screening following the best-practice guidelines outlined on the R/qtl website (http://www.rqtl.org/tutorials/geneticmaps.pdf). We removed all individuals with genotypes for fewer than 50% of markers, and all markers with genotypes for fewer than 50% of individuals. We additionally screened for uninformative markers with duplicate information, and any markers showing extreme segregation distortion (*i.e.*, being nearly monomorphic). We then estimated a genetic map using the *est.rf* function. Following previously published information on *C. lectularius* karyotype (Sadílek *et al.* 2013), showing an average of 14 autosome pairs and one X chromosome, we varied the maximum recombination fraction and the minimum LOD score (*i.e.*, “logarithm of the odds score”—a log_10_ transformation of the likelihood ratio between a model with linkage and a null model) threshold in order to create approximately the same number of LG as autosomes. We then checked our initial genetic map following the R/qtl guidelines (http://www.rqtl.org/tutorials/geneticmaps.pdf), and removed any problematic markers before reordering markers based on likelihood analysis of permuted marker orders using the *ripple* function. The R script used to produce our genetic map is available at Dryad (http://dx.doi.org/10.5061/dryad.d4r50).

Following map construction, we performed standard interval mapping with a single QTL model for pesticide resistance using R/qtl. In order to account for genotyping error in our QTL analysis, we applied a maximum likelihood-based estimate of error rate. QTL genotype probabilities were then calculated using a Kosambi mapping function, and an error probability based on our maximum-likelihood estimation. We then used the *scanone* function to estimate QTL LOD scores using both the EM algorithm and Haley-Knott regression. To test the significance of our QTL, and to estimate confidence limits on QTL positions, we reanalyzed our dataset with *scanone* using 1000 permutations.

### Testing for sex-linkage

A limitation of our *F*_2_ mapping approach was that it did not allow for mapping of putatively sex-linked loci. Furthermore, as *F*_2_ individuals were phenotyped and processed for DNA extraction as 4th instar nymphs, we were unable to determine their sex. In order to account for potential sex-linkage in our RAD dataset, we identified loci that were heterozygous in the female *F*_1_ parent, and homozygous for the grandmother’s allele in the male *F*_1_ (*i.e.*, AB/AA). Our rationale for this was that, assuming no error, a cross using AA × BB grandparents should result in only homozygous genotypes for loci that occur on the sex chromosome in the heterogametic sex. To rule out error, we focused only on loci with an AA genotype in the female grandparent and with >50% of individuals genotyped. Using this set of putatively sex-linked loci, we then performed a Chi-squared test of independence to test for an association with the resistance phenotype. False Discovery Rate (FDR) correction was used to account for multiple testing; since many loci are not independent (*i.e.*, multiple loci map to the same scaffold), we used the number of scaffolds, and the minimum *P*-value for each scaffold, to perform this correction.

### Identifying candidate genes in functional regions

RAD-seq loci are typically short (*i.e.*, ∼100 bp), and, since they sample only a relatively small proportion of the genome, are unlikely to occur within resistance genes themselves. Similarly, short consensus RAD loci are unlikely to be of much use in identifying candidate genes using a functional analysis such as BLAST. To identify candidate genes associated with QTL regions, we first used the calculated 95% Bayesian credible intervals around the QTL. Using the markers flanking the interval, we then located the corresponding physical position in the reference genome, and identified all candidate genes within this interval. We also searched for genes associated with pyrethroid resistance on the same scaffold as our identified QTL. Genes were identified from recently published annotations (Benoit *et al.* 2016), and extracted using custom R scripts.

### Genome reassembly

In order to combine our genetic map with the recently published *C. lectularius* reference genome, we used Chromonomer (Amores *et al.* 2014). Chromonomer first removes markers that are inconsistent with local assembly order on the genetic map, and then anchors genome scaffolds to LG based on marker mapping position before finally reassembling the genome accordingly. Chromonomer was run using the default settings as described in the online manual (http://catchenlab.life.illinois.edu/chromonomer/manual/).

### Data availability

Raw RAD-sequencing reads are archived at EMBL-ENA (PRJEB15267). All bash scripts for alignment, filtering, trimming and Stacks catalog construction are archived on Dryad (http://dx.doi.org/10.5061/dryad.d4r50). All R scripts for R/qtl analysis are also archived on Dryad. The reassembled genome is archived in NCBI GenBank (GCA_000648675.2), and is hosted at https://i5k.nal.usda.gov/Cimex_lectularius.

## Results

### RAD sequence mapping and Stacks catalog construction

Following filtering for quality and PCR duplicates, an average of 240,898 ± 150,591 (mean ± SD) reads was retained for each individual (see Table S1). A high proportion of these reads (78.0% ± 5.61) mapped to the reference genome (see Table S1). Initial RAD locus catalog construction resulted in 12,992 unique RAD loci, which was reduced to 12,962 tags following *rxstacks* correction. Of these corrected RAD loci, 1171 occurred in >8 of the *F*_2_ progeny; 430 of these loci were heterozygous in both *F*_1_ parents, and were subsequently included for genetic map construction.

### Genetic map construction

Prior to map construction, we filtered individuals with a high proportion of missing markers, markers missing in a high proportion of individuals (both >50%), duplicate markers (*i.e.*, likely originating from either side of the same RAD locus), and markers with a highly distorted segregation ratio. This resulted in a reduced dataset of 75 individuals and 357 high quality markers.

**Figure 1:**
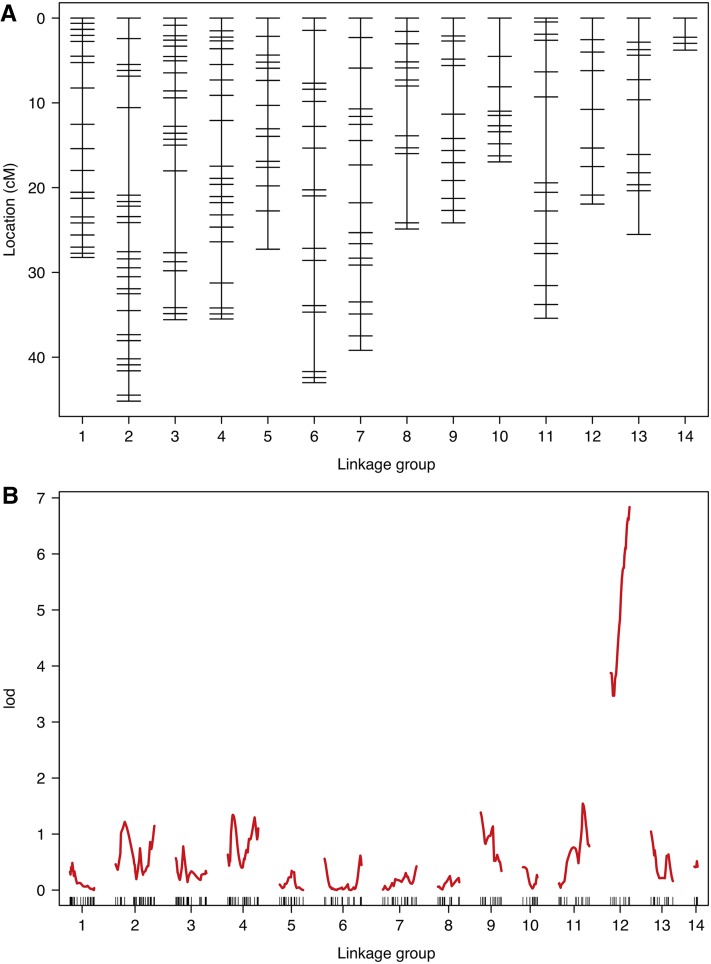
Linkage map and QTL analysis. (A) Linkage map showing positions of SNP markers for *C. lectularius F*_2_ cross over 14 inferred LG (putative chromosomes). (B) LOD scores for markers across the genome reveals a strong and significant peak on LG 12.

Initial recombination fraction estimates were strongly correlated with high LOD scores (Figure S1). To account for this, we merged markers into 31 LG; *i.e.*, ∼2 LG per chromosome (assuming *n* = 15), representing correct, and potentially misidentified, alleles. LG were combined based on high LOD scores, but low recombination fractions among markers (Figure S2). Following additional filtering, allele correction, and removal of loci with apparent genotyping errors and/or extreme segregation distortion, we re-estimated LG to ensure high LOD, and low recombination fractions, among markers on the same chromosome (Figure S3). Our final map, based on 71 individuals after filtering, was 407 cM long, with an average spacing of 1.3 cM between each of the 334 markers and consisted of 14 LG (Figure 1A, Figure S3, and Table 1). Scaffold positions of all mapped markers are given in Table S2. Given the genome size of 650.5 Mb, this implies an average recombination rate of 0.6 cM/Mb.

**Table 1:** Genetic map summary

LG	No. Markers	Length (cM)	Length (Mb)	Mean Spacing (cM)	Max Spacing (cM)
1	38	28.23	39.95	0.76	4.28
2	37	45.18	48.13	1.26	10.32
3	34	35.58	34.27	1.08	9.66
4	33	35.49	54.64	1.11	5.39
5	30	27.26	49.12	0.94	4.52
6	27	43.01	32.09	1.65	7.01
7	25	39.19	44.27	1.63	4.81
8	21	24.88	30.64	1.24	8.17
9	19	24.15	22.31	1.34	5.74
10	19	16.96	13.04	0.94	4.52
11	18	35.41	38.21	2.08	10.13
12	15	21.93	16.06	1.57	4.57
13	14	25.53	6.51	1.96	6.45
14	4	3.78	4.48	1.26	2.26
All	334	406.58	433.71	1.27	10.32

Summary of marker number, spacing, map distance, and physical size (from reassembled genome) for *C. lectularius* LG.

### QTL analysis

Maximum-likelihood estimation indicated genotyping error rate was 0.0025, suggesting such error was not an issue in our filtered mapping dataset (Figure S4). Per-locus estimates of error rates suggested few consistent errors across loci, therefore, QTL analysis was first conducted without manual genotype correction. QTL scans for pesticide resistance using both the Hayley-Knott and EM algorithms revealed a clear signal of a single QTL toward the end of LG 12, centered on RAD locus r449_NW_014465016 (LOD = 6.84, *P* = < 0.0005 based on 10,000 permutations; see Figure 1B). No genotyping errors were present on this LG, and repeating this analysis with manual corrections produced identical results. Examining phenotype counts at this locus clearly showed that AA homozygotes showed complete pesticide susceptibility, whereas 90% of BB homozygotes were resistant, and 10% partially resistant (Table 2). Heterozygotes at this locus were mainly susceptible (66%), although some showed partial resistance (24%), and a minority showed full resistance (10%, see Table 2). The LG12 QTL explained 64.2% of the variation in phenotype, indicating pesticide resistance is not completely explained by this bi-allelic QTL.

**Table 2:** Genotype-phenotype counts and percentages at the focal marker (r2020_s2) on chromosome 12

Genotype	Partial Resistance	Resistance	Susceptible
AA	0	0	22 (100.0%)
AB	7 (24.1%)	3 (10.3%)	19 (65.5%)
BB	2 (10%)	18 (90.0%)	0

### Sex-linkage

Identifying loci that were AB/AA in the *F*_1_ cross, and with an AA genotype in the grandmother, resulted in 106 putatively sex-linked loci that were not included in our genetic map construction. Chi-squared tests for independence identified four out of the 106 putatively sex-linked loci occurring on different scaffolds that showed an association with the resistance phenotype (*P* < 0.005; Table S3), but none of these associations remained significant following FDR correction.

### Candidate gene identification

Our pyrethroid resistance QTL maps to Scaffold 2 (start position = 15,333,169) of the reference genome. The LOD peak surrounding this QTL on LG12 of our linkage map spanned a total 95% Bayesian credible interval of 11.1 cM (*i.e.*, from 10.8 to 21.9 cM). This corresponds to the last ∼6.8 Mb of scaffold 2, and the last ∼4 Mb of scaffold 18 in the reference genome (*N.B*. our linkage map suggests a reverse orientation for some scaffolds, including scaffold 18). Searching for coding sequences within these scaffold intervals, we identified 211 unique gene names (Table S4). The most likely candidate for pyrethroid resistance was an ubiquitin carboxyl-terminal hydrolase 15-like gene occurring 1.2 Mb downstream from our QTL peak. However, we also identified two other putative candidates occurring further downstream (*i.e.*, >3 Mb) on scaffold 2: a VSSC protein para gene and a glutathione S-transferase (see Table 3). Additionally, we identified a cytochrome P450 6B5-like gene on Scaffold 18 (Table 3). Finally, three of the four putatively sex-linked RAD-loci that showed an association with resistance, mapped to scaffolds containing three further candidate gene classes: a glutathione-S transferase (Scaffold 6), a cuticular protein gene cluster (Scaffold 24), and P450 genes (Scaffold 31).

**Table 3:** Putative pyrethroid resistance candidate genes

Gene name	NCBI Gene ID	Scaffold	Start (bp)	End (bp)
Ubiquitin carboxyl-terminal hydrolase 15-like	106668434	Scaffold 2	16,617,167	16,638,289
Sodium channel protein para	106667833	Scaffold 2	18,435,119	18,463,718
Glutathione S-transferase	106666926	Scaffold 2	21,129,642	21,130,758
Cytochrome P450 6B1-like	106663981	Scaffold 18	3,480,876	3,508,711
Cytochrome P450 6B1-like	106663982	Scaffold 18	3,456,406	3,461,345
Cytochrome P450 6B5-like	106663983	Scaffold 18	3,404,836	3,440,903
Probable cytochrome P450 6a14	106663984	Scaffold 18	3,476,053	3,495,873

Gene name, original reference genome scaffold, and position for putative pyrethroid resistance genes identified in LG12 resistance QTL 95% Bayesian probability interval.

### Genome reassembly

Chromonomer pruned 130 markers from our genetic map that were inconsistent with local assembly order, using 208 well-behaved markers to perform genome reassembly. Of the 1402 scaffolds in the previously published reference genome, 69 were anchored to our linkage map, whereas five aligned to more than one position and were split, resulting in a total of 74 anchored scaffolds (mean size: 7.5 Mb, range: 0.06–33 Mb). Chromonomer was thus able to reassemble 67% of the genome into 14 autosomal LG spanning 433 Mb. The newly reassembled genome (GCA_000648675.2) is available at https://i5k.nal.usda.gov/Cimex_lectularius.

## Discussion

We constructed the first linkage map for the common bed bug, *C. lectularius*, by performing an *F*_2_ insecticide resistance mapping cross using 334 high quality SNP markers identified with RAD-sequencing. Our final linkage map consisted of 14 putative LG, and was 407 cM in length, with an average marker spacing of 1.3 cM. We successfully demonstrated the ability of the linkage map to order scaffolds from the newly available bed bug genome by anchoring 74 scaffolds to linkage map positions. Therefore, we were able to reassemble 67% of the draft genome into our putative LG, facilitating refinement and validation of the current genome assembly. In addition to constructing a genetic map, we detected a biallelic QTL on LG 12 that explains 64% of variation in pyrethroid resistance, in very close proximity (1.2 Mb) to a carboxylesterase encoding candidate gene for pyrethroid resistance (Zhu *et al.* 2013; Benoit *et al.* 2016), and <10 cM (but still within the 95% Bayesian credible interval) from the VSSC, another candidate strongly associated with insecticide survival in other studies (Yoon *et al.* 2008, Zhu *et al.* 2010).

Our construction of a genetic map for *C. lectularius* should be considered a first attempt to assemble the recently published reference genome into clusters of markers related by linkage. We stress that our map estimates only LG and genetic distance. Its relationship to the actual physical map of the *C. lectularius* genome remains uncertain, because a considerable proportion of the genome remains unassembled into any LG (∼33%). Importantly, this also includes the sex chromosome, which we were unable to map due to our cross design, although we did identify putatively sex-linked scaffolds (Table S3).

Bed bugs, like other Cimicidae, have received attention for their unusual cytogenetic characteristics (*e.g.*, Darlington 1939; Slack 1939; Ueshima 1967; Grozeva *et al.* 2010, 2011). For example, one study showed that populations appeared to be geographically variable for their karyotype across Europe, with 2*n* chromosome number varying from 29 to 47, which was further complicated by fragmentation of sex chromosomes in some populations (Sadílek *et al.* 2013). Since the grandparents from our cross were not karyotyped, the expected number of chromosomes in our *F*_2_ generation is unknown, and may even be variable among individuals. Excluding sex chromosomes, and assuming 2*n* = 28 autosomes, we would expect to identify at least 14 LG in our analysis. Therefore it seems likely that the majority of our LG correspond to physical autosomes. Due to our cross design, we were unable to ascertain the sex of *F*_2_ individuals, preventing us from including sex as a mappable trait, or from clearly identifying sex-linked loci. Furthermore, by only including loci heterozygous in both *F*_1_ parents (*i.e.*, using an AB × AB cross), we were also unable to identify LG putatively associated with sex. However, using an independent analysis outside of our linkage map construction, we were able to identify a proportion of potentially sex-linked loci, and, by extension, genome scaffolds that may anchor to the sex chromosome. Additional crosses are necessary to identify genomic regions specifically involved in sex-determination. Nonetheless, further work, such as FISH-based mapping, is now possible, and necessary, to physically map our inferred LG to *C. lectularius* chromosomes.

Using QTL mapping, we identified a clear signal of a single biallelic QTL related to pyrethroid resistance on LG12, with all AA genotypes completely susceptible, all BB genotypes showing resistance, or at least partial resistance, and 66% of heterozygotes being susceptible. These proportions suggest our QTL is partly recessive.

Given the large LOD peak confidence intervals, it is unclear whether the QTL identified here represents the actions of a single gene, or a complex of multiple coadapted genes for pyrethroid resistance. Importantly, the resistance QTL occurs in close proximity to several previously identified candidate genes for pyrethroid resistance. This suggests that, despite our relatively high-density approach using reduced-representation sequencing, we did not have adequate resolution to identify the exact candidate gene involved in insecticide resistance. Additional higher resolution QTL mapping, using a combination of high and low coverage whole-genome resequencing of larger families may allow more fine scale identification of the exact resistance QTL in this context (Glazer *et al.* 2015). Despite this, our RAD-seq QTL analysis has identified a region containing several important known candidate genes for pyrethroid resistance.

The first and closest of these candidates, a ubiquitin carboxyl-terminal hydrolase 15-like gene occurs just 1.5 Mb from our inferred QTL. Carboxylesterases are a gene family coding for esterase enzymes that hydrolyze ester bonds present in a wide variety of insecticides, including pyrethroids (Montella *et al.* 2012). More efficient metabolic breakdown, resulting in a decrease in insecticide concentration following exposure has previously been implicated as a means of pyrethroid resistance in bed bugs (Zhu *et al.* 2013). Metabolic breakdown genes are likely to contribute to insecticide resistance via at least one of three mechanisms: (1) gene duplication, (2) increased gene expression, or (3) mutation in the enzyme-coding sequence (Montella *et al.* 2012). Gene annotation reveals at least 30 carboxylesterase genes in the *C. lectularius* genome, with clustering on some scaffolds (Benoit *et al.* 2016). Furthermore, an expression analysis of geographically widespread bed bug populations indicated overexpression of a carboxylesterase gene in resistant samples (Zhu *et al.* 2013). However, the cluster of carboxylesterase genes found on genome scaffold 18 does not map to LG 12. In addition, the carboxylesterase candidate gene close to the LOD peak of our QTL differs from the overexpressed gene reported previously. The LG12 QTL may therefore represent a gene that is not overexpressed, *e.g.*, a transcription factor involved in expression regulation of multiple carboxylesterase genes. Additionally, our QTL explains ∼64% of the variation in resistance phenotypes, meaning that other genes may be involved. Further investigation is required to examine whether coding mutations in the ubiquitin carboxyl-terminal hydrolase 15-like gene on genome scaffold 2 may result in more efficient metabolic breakdown of pyrethroid insecticides.

In addition to the ubiquitin carboxyl-terminal hydrolase 15-like gene, the QTL is located upstream of two other major pyrethroid resistance candidates, the VSSC and the metabolic detoxifying enzyme coding glutathione S-transferase gene. Knockdown (*kdr*) resistance to pyrethroids is increasingly widespread in bed bugs (*e.g.*, Zhu *et al.* 2010), with *kdr* mutations at the target site of pyrethroids, the VSSC, identified as major mechanism for resistance (Yoon *et al.* 2008). However, there is increasing evidence for a more complex basis for this trait, with penetrative (Mamidala *et al.* 2012; Zhu *et al.* 2013) and metabolic mechanisms (Zhu *et al.* 2012), as well as behavioral avoidance (Romero *et al.* 2009), associated with pyrethroid resistance. This is not a unique feature of bed bugs, with evidence of interactions between multiple insecticide resistance mechanisms found in a number of medically and economically important pests, *e.g.*, German cockroach (Anspaugh *et al.* 1994), cotton bollworm (Martin *et al.* 2002), houseflies (Georghiou 1969, Sawicki, 1970, Shono *et al.* 2002), and mosquitoes (Perera *et al.* 2008; Awolola *et al.* 2009; Hardstone *et al.* 2009). These and our results, therefore, support the view that understanding the interaction between resistance loci will be an important part of developing new resistance management strategies (Hardstone *et al.* 2009). For example, epistatic interactions between resistance loci (*e.g.*, Bohannan *et al.* 1999) may reduce their costs (Gordon *et al.* 2015), facilitating the maintenance and spread of resistant alleles. Interestingly, the QTL identified in the present study occurs in close proximity to genes coding for detoxifying metabolic enzymes, as well as the VSSC. Future work should focus on identifying the causal mutation(s) underlying this QTL, and how they interact with previously identified resistance loci in bed bugs.

In addition to multiple resistance mechanisms, bed bug metapopulation structure (Fountain *et al.* 2014) may further promote the spread of resistance alleles. For example, if an insecticide-resistant individual enters a (usually inbred; Fountain *et al.* 2014; Booth *et al.* 2015) bed bug population, both heterosis (Fountain *et al.* 2015), and the introduction of resistance alleles (Saccheri and Brakefield 2002; Song *et al.* 2011), may lead to the rapid recovery of a population and spread of resistance. Rapid selection to environmental disturbance can be prevalent in metapopulations (Reznick and Ghalambor 2001; Bell and Gonzalez 2011), and this may also have contributed to the rapid spread of resistance mutations in bed bug populations both in the United States (Zhu *et al.* 2010) and Europe (Booth *et al.* 2015).

To conclude, our mapping cross identified a QTL in close proximity to a number of candidate genes related to pyrethroid resistance, and thereby provides strong evidence that these candidate genes play a major role in determining survival following pesticide treatment. Functional assays and higher resolution QTL approaches should now investigate the exact mechanism by which these genes convey resistance. The recent availability of the bed bug genome (Benoit *et al.* 2016; Rosenfeld *et al.* 2016), complete with a full list of potential candidate genes related to insecticide resistance, in addition to the linkage map generated here, will provide an excellent resource for future research on the development and spread of insecticide resistance in the bed bug.

## Supplementary Material

Supplemental Material
