# Supplementary material for: A Linkage Map and QTL Analysis for Pyrethroid Resistance in the Bed Bug Cimex lectularius
Source: G3 (Bethesda). 2016 Oct 12;6(12):4059–66. doi: 10.1534/g3.116.033092 (PMC5144974; doi:10.1534/g3.116.033092)
Supplement: Supplemental Material [file supp_6_12_4059__index.html]

A Linkage Map and QTL Analysis for Pyrethroid Resistance in the Bed Bug Cimex lectularius — A Linkage Map and QTL Analysis for Pyrethroid Resistance in the Bed Bug Cimex lectularius — Supplemental Material 

# A Linkage Map and QTL Analysis for Pyrethroid Resistance in the Bed Bug *Cimex lectularius*

## Supplemental Material for Fountain *et al.*, 2016

**Files in this Data Supplement:**

- Figure S1 - LOD scores vs. recombination fraction prior to correction. (.pdf, 7 MB)
- Figure S2 - High LOD scores and low recombination fractions. (.pdf, 15 KB)
- Figure S3 - LOD scores vs. recombination fraction following correction and filtering. (.pdf, 471 KB)
- Figure S4 - Maximum-likelihood estimation of genotyping error rate. (.pdf, 5 KB)
- Table S1 - Read statistics. (.csv, 4 KB)
- Table S2 - Marker information: Reference genome scaffold, left-most base pair position (i.e. alignment start position), linkage group and physical map position for RAD markers used in genetic map construction. (.csv, 15 KB)
- Table S3 - Putative sex-linked loci and mapped scaffolds. (.csv, 337 B)
- Table S4 - Genes located in pyrethroid resistance QTL regions. (.csv, 20 KB)
