# Supplementary figures and images for: A Linkage Map and QTL Analysis for Pyrethroid Resistance in the Bed Bug Cimex lectularius
Source: G3 (Bethesda). 2016 Oct 12;6(12):4059–66. doi: 10.1534/g3.116.033092 (PMC5144974; doi:10.1534/g3.116.033092)

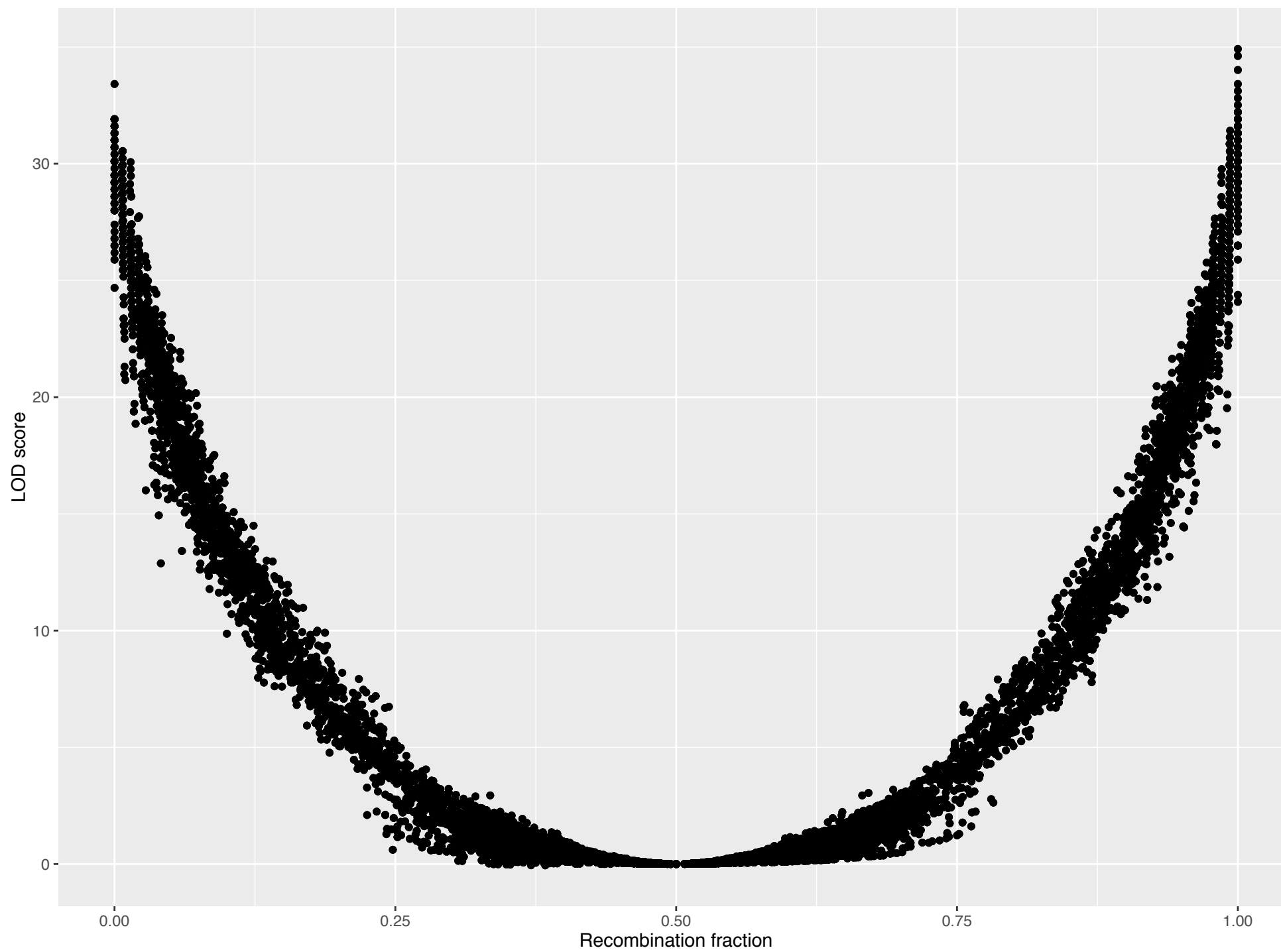

Supplement: Supplemental Material [file supp_g3.116.033092_FigureS1.pdf]

**r1697\_NW\_014465022**

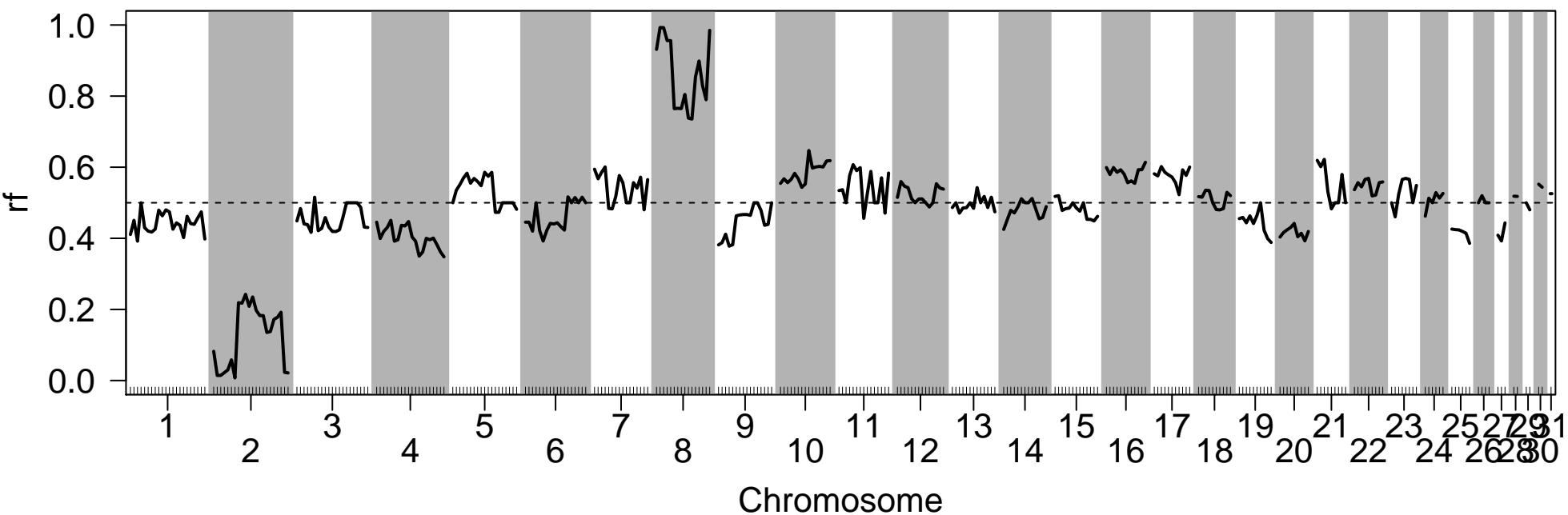

**r1697\_NW\_014465022**

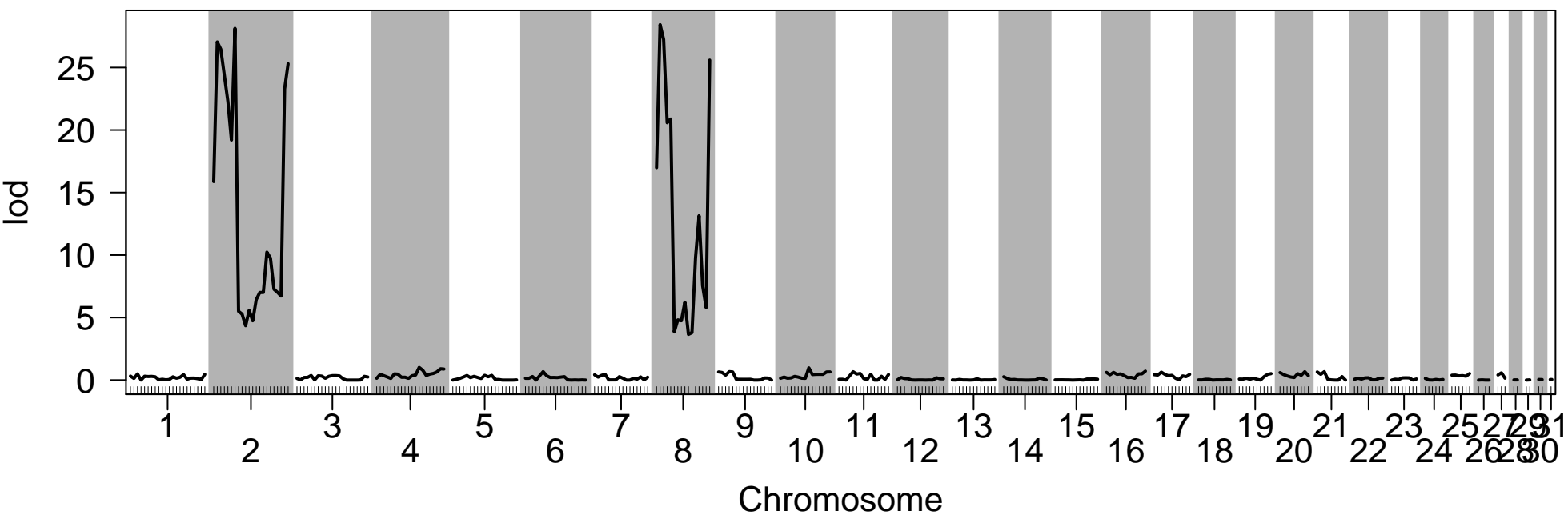

Supplement: Supplemental Material [file supp_g3.116.033092_FigureS2.pdf]

1 2 3 4 5 6 7 8 9 10 11 12 13 14

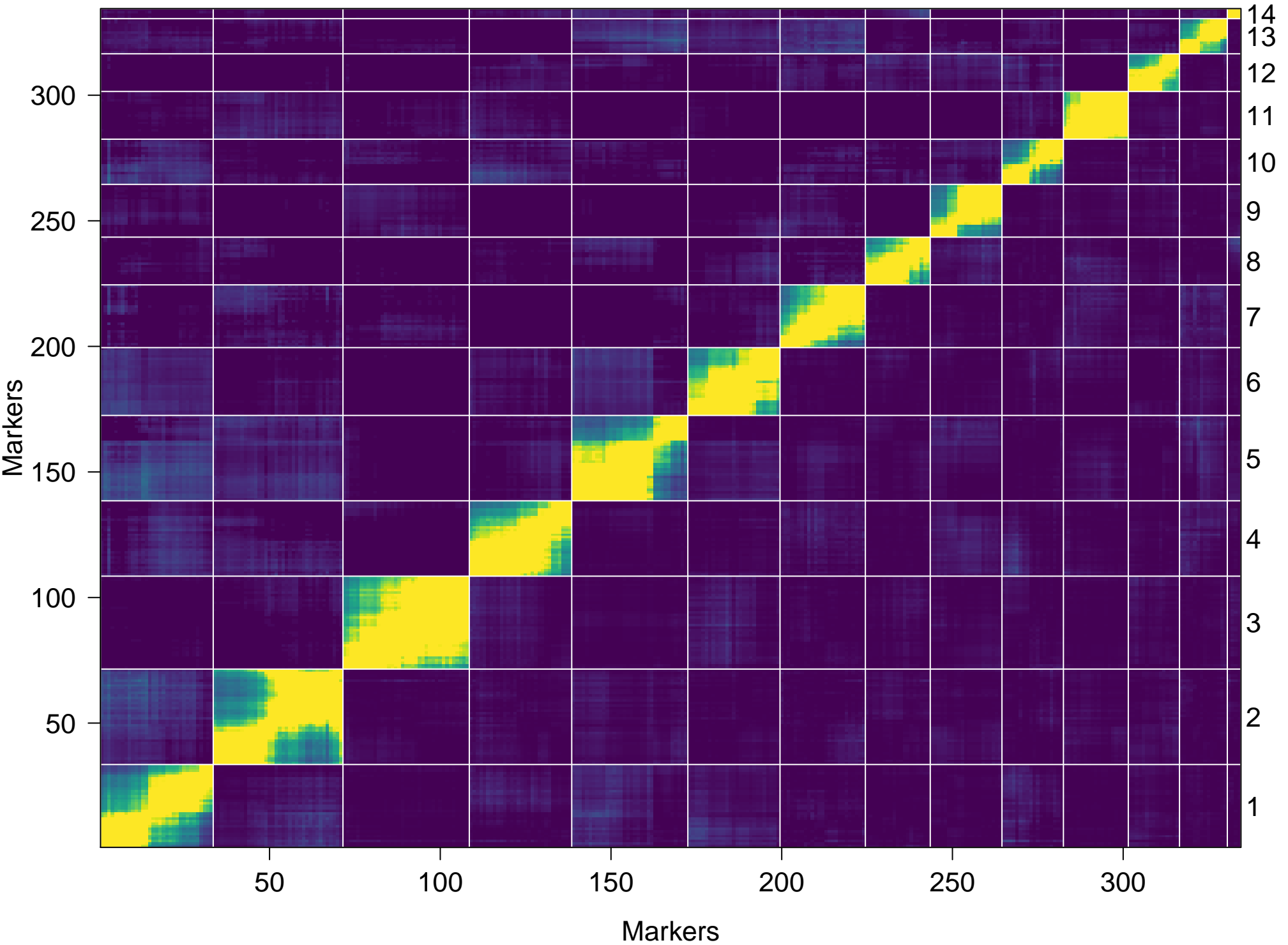

Supplement: Supplemental Material [file supp_g3.116.033092_FigureS3.pdf]

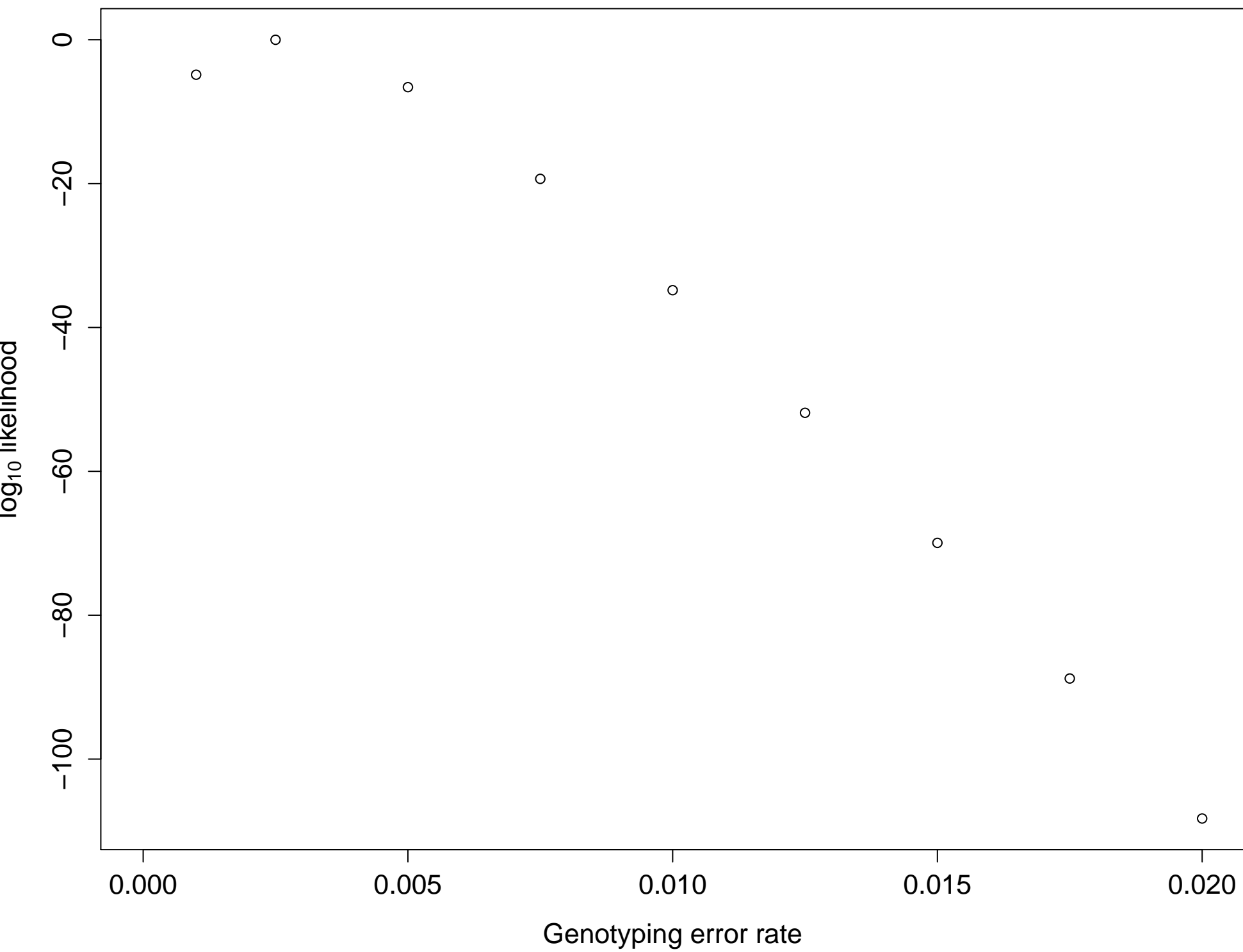

Supplement: Supplemental Material [file supp_g3.116.033092_FigureS4.pdf]
